# Comprehensive Analysis of the COVID-19: Based on the Social-Related Indexes From NUMBEO

**DOI:** 10.3389/fpubh.2022.793176

**Published:** 2022-04-28

**Authors:** Xuecan Guo, Ruiyu Chai, Yan Yao, Yanbiao Mi, Yingshuang Wang, Tianyu Feng, Junwei Tian, Bocheng Shi, Jiwei Jia, Siyu Liu

**Affiliations:** ^1^Department of Epidemiology and Biostatistics, School of Public Health, Jilin University, Changchun, China; ^2^Department of Computational Mathematics, School of Mathematics, Jilin University, Changchun, China; ^3^Jilin National Applied Mathematical Center, Jilin University, Changchun, China

**Keywords:** COVID-19, time series analysis, social-related Indexes, climate, k-means clustering algorithm

## Abstract

**Background:**

The COVID-19 has been spreading globally since 2019 and causes serious damage to the whole society. A macro perspective study to explore the changes of some social-related indexes of different countries is meaningful.

**Methods:**

We collected nine social-related indexes and the score of COVID-safety-assessment. Data analysis is carried out using three time series models. In particular, a prediction-correction procedure was employed to explore the impact of the pandemic on the indexes of developed and developing countries.

**Results:**

It shows that COVID-19 epidemic has an impact on the life of residents in various aspects, specifically in quality of life, purchasing power, and safety. Cluster analysis and bivariate statistical analysis further indicate that indexes affected by the pandemic in developed and developing countries are different.

**Conclusion:**

This pandemic has altered the lives of residents in many ways. Our further research shows that the impacts of social-related indexes in developed and developing countries are different, which is bounded up with their epidemic severity and control measures. On the other hand, the climate is crucial for the control of COVID-19. Consequently, exploring the changes of social-related indexes is significative, and it is conducive to provide targeted governance strategies for various countries. Our article will contribute to countries with different levels of development pay more attention to social changes and take timely and effective measures to adjust social changes while trying to control this pandemic.

## Introduction

The outbreak of COVID-19 has been accompanied by an exponential increase of new infections and a growing death count. The WHO has reported over 440 million diagnosed infections, and more than 5.98 million patients lost their lives worldwide as of 6 March 2022 (1). This sudden pandemic has hit the medical assistance systems of many countries hard and has disrupted the normal public order (2).

Many countries made different containment and targeting strategies (3), and country-based mitigation measures will influence the occurrence and development of COVID-19 (4). When it comes to public hygiene interventions, Hong Kong, which is more liberal, trends to voluntary and stepwise action instead of implemented strict compulsory testing and isolation (5). Meanwhile, the Austrian government is revising and submitting a bill to force all residents to receive COVID-19 vaccine in order to reduce the risk of infection and the spread of the disease (6). The Changning District of Shanghai has mainly adopted some strategies to prevent and control COVID-19, such as focusing on key populations and key areas, standardizing the workflow, and investigation and closed-loop management to control new cases (7). The Ethiopian government made tremendous efforts to control COVID-19, such as limiting public gathering, closing borders, and restricting transportation (8). Certainly, the degrees of severity of the pandemic in different countries vary. It is mostly dependent on government policies, detection intensity, mass awareness of prevention, vaccination status, medical facilities, traffic propagation rate, and supervision (9–11).

As one of the most severe pandemics in the last eight decades, there is a link between the grave circumstances of COVID-19 and diverse societal levels. The impact of COVID-19 on the economy should be recognized. There is a drastic effect on the worldwide economy, with an estimated loss of more than 1 trillion dollars (12). Some researchers claimed that the effect of this pandemic on sector fluctuations was greater than the global financial crisis (13). In terms of quality of life, Chinese researchers confirmed that COVID-19 has aggravated mild stress, but improved social and family support for residents (14). Research on Korean adults shows that their lifestyle has changed and that their daily activities are restricted. In addition, the quality of life and mental health declined (15). The epidemic has a greater impact on children and adolescents in Germany, especially minors in families with difficult domestic life or immigrant background (16). The control of COVID-19 is also closely related to the medical supplies and treatment services. The rapid establishment of the Fire God Mountain hospital and the Thunder God Mountain hospital had provided patients with timely suitable rescue and treatment environments in Wuhan, China (17). The challenges and pressing actions for the United States especially highlights the problem of continuous improvement and optimization of the supply chain of health care in the United States (18). In addition to reducing disease transmission, COVID-19 mitigation strategies have also reduced urban road traffic, resulting in indirect benefits air quality, traffic noise, and accidents (19). To slow down the transition, most countries recommended that people reduced aggregation activities. Some countries even released prisoners by means of parole and probation. However, it would lead to a degree of destruction of social security and stability (20).

We select nine social-related indexes from NUMBEO. These indexes include quality of life index, climate index, health care index, safety index, cost of living index, and so forth. We utilize the time series model (21). The differences between the forecast and actual values of 2020 mid-year are predicted and compared. The D-value (the forecast of 2020 mid-year minus actual values of 2020 mid-year) of nine indexes are calculated. The cumulative incidence (CuI) and the score COVID-safety-assessment (SCSA) of selected country are collected. Correlation analyses between these two variables and the D-value are performed. It is valuable that we analyze the social conditions of many countries from a macro perspective about COVID-19. Countries with different development levels are facing diverse situations. No matter to what extent the COVID-19 is, the impacts of COVID-19 and the changes in social-related aspects should not be ignored. We aim to find out which index in countries is more affected by COVID-19. This will help countries with different levels of development pay more attention to social changes and take timely and effective measures to adjust social changes while trying to control this pandemic. Moreover, social stability is more conducive to the implementation of epidemic prevention and control work.

## Materials and Methods

### Nine Indexes of 52 Countries

We first searched for relevant social news and relevant social indicators through Google, Baidu, and other websites. After information screening, we initially selected the NUMBEO website. NUMBEO was quoted by numerous newspapers, magazines, and blogs [e.g., Time, BBC, People (China, in Chinese), and so on]. Then, after consulting relevant literature, we finally decided to select the NUMBEO website (22, 23). NUMBEO is the world's largest cost of living database. It is also a crowd-sourced global database of quality-of-life information that includes perceived crime rates, quality of health care, and pollution index, among many other statistics. The detailed introduction of each index was shown in Supplementary Table 1. It contributed data about cities and countries worldwide. The data provided by the website is public and can be downloaded directly without processing. We collected data published from 2014 to 2020 (24). After deleting countries with nine missing indexes and combining the national information provided in the data of SCSA, we finally selected 52 countries, including nine indexes, and SCCA data.

### Cumulative Incidence

The cumulative confirmed number of COVID-19 cases of 52 countries were obtained from Netease website from the first reported case to 1 July 2020 (25). The data from the Netease website were from official and media reports of various countries and regions. We collected the total population of the selected countries (26) to calculate cumulative incidence that indicated the severity of the epidemic.


$$\begin{array}{l} \text{CuI}=\frac{\text{number of new cases }}{\text{total populaition}}\times 100\% \end{array}$$

### SCSA

The data of SCSA was from the COVID-19 Regional Safety Assessment. It included Big Data Analysis of 200 Countries, Regions, and Territories that were published by the Deep Knowledge Group (27). The SCSA was based on the analysis of 130 quantitative and qualitative parameters and 11,400 data from 200 COVID-19 endemic countries around the world in June by the deep knowledge group.

### D-Value

The D-value of nine indexes is the forecast of 2020 mid-year minus actual values of 2020 mid-year.

D-value = (the forecast values of 2020 mid-year) - (the actual values of 2020 mid-year).

### Statistical Methods

#### Time Series Models

The time series model tries to predict unknown data by modeling historical surveillance data (28–30). To solve the problem of data with a long time span and small quantity, several time series models, including the naive, simple average, and exponential smoothing, were employed in this study. The Naive method is suitable for data with high stability. The simple average method fits data with more stability. The exponential smoothing is suitable for forecasting data with no trend or seasonal pattern. A major advantage of these three methods was that they were suitable for processing simple and stable data in line with the characteristics of the data we collected (Supplementary Figure 1 and Supplementary Table 2). The mathematical formulas were expressed as follows:


$$\begin{array}{l} {\text{F}}_{\text{t}1}={\text{A}}_{\text{t}1-1}, \end{array}$$

where F_t1_ is the forecast value at the t moment and A_t1−1_ is the actual value at the moment of t1-1;


$$\begin{array}{l} {\text{F}}_{\text{t}2}=\frac{\sum_{\text{i}=1}^{\text{n}}{\text{A}}_{\text{t}2-\text{i}}}{\text{n}}, \end{array}$$

where F_t2_ is the forecast value at the t2 moment, i is the corresponding moment, n is the number of data, and A_t2−1_ is the actual value at the moment of t2-1;


$$\begin{array}{l} {\text{F}}_{\text{t}3}={\text{F}}_{\text{t}3-1}+\text{α}\left({\text{A}}_{\text{t}3-1}-{\text{F}}_{\text{t}3-1}\right), \end{array}$$

where F_t3_ is the forecast value at the t moment, A_t3−1_ is the actual value at the moment of t3-1, F_t3−1_ is the forecast value at the moment of t3-1, A_t3−1_ is the actual value at the moment of t3-1, and α smoothing coefficient whose value is between 0 and 1.


$$\begin{array}{l} \text{RMSE}=\sqrt{\frac{1}{\text{n}}\sum_{\text{t}=1}^{\text{n}}{\left({\text{F}}_{\text{t}}-{\text{A}}_{\text{t}}\;\right)}^{2}}, \end{array}$$

Based on the data of nine indexes of 52 countries from 2014 to 2019, we used three methods to predict 2020 data. By comparing the actual and forecast value in 2020, we selected the optimal model according to the root mean squared error (RMSE) to forecast the corresponding value in mid-year of 2020. Through comparing the difference between forecast and actual values in mid-year 2020, we used a paired-sampled *T*-test to find the indexes that were affected during COVID-19 pandemic.

#### K-Means Clustering Algorithm

To cluster the 52 countries and further explore the impact of COVID-19 on social-related indexes, we used the k-means clustering algorithm. The variables used to create patterns were the differences among these nine indexes and whether the countries were developed.

#### Bivariate Statistical Analysis

We used the parametric test, Mann-Whitney U-test, and Pearson correlation analysis to find the indexes affected by COVID-19. All statistical analysis were performed by the Python (Version 3.7.9). The significance level set with *p*-value < 0.05.

## Results

### Forecasting the Nine Indexes Using the Naive Model

The RMSE values of different methods were compared to judge the error of prediction effect. The naive method showed the best RMSE value. Hence, the naive method was the best model among the three time series models for our data. The results of RMSE of three methods are shown in Table 1. We used the naive model to forecast the nine indexes of the 52 countries (Supplementary Figure 1 and Supplementary Table 2). It could be observed from Table 2 that there was no significance between the actual and forecast value in 2020 of Climate Index (CI), Cost of Living Index (CLI), Health Care Index (HCI), Pollution Index (PI), and Traffic Commute Time Index (TCTI). Therefore, it can be said that COVID-19 has little impact on these indexes. There was significance in Quality-of-Life Index (QLI), Property Price to Income Ratio Index (PPIRI), Purchasing Power Index (PPI), and Safety Index (SI), indicating that during the COVID-19 pandemic, QLI, PPI, and SI were decreased, while PPIPI was increased.

**Table 1:** Error measures obtained under the three time series models.

**Index**	**Naive method**	**Simple average method**	**Simple exponential smoothing method**
Quality of life index	2.52	4.36	2.64
Climate index	0.87	4.15	1.08
Cost of living index	1.45	3.17	1.46
Health care index	1.04	1.79	1.07
Pollution index	1.27	2.18	1.28
Property price to income ratio index	0.96	1.66	0.98
Purchasing power index	3.48	4.94	3.81
Safety index	1.26	2.14	1.34
Traffic commute time index	1.56	2.33	1.57

**Table 2:** The results of paired Student's *T*-test for each index.

**Index**	***t***	***p***
Quality of life index^*^	−4.76	1.65e^−05^
Climate index	−0.98	0.33
Cost of living index	−0.13	0.90
Health care index	0.78	0.44
Pollution index	0.83	0.41
Property price to income ratio index^*^	2.26	0.03
Purchasing power index^*^	−11.90	2.44e^−16^
Safety index^*^	−2.94	0.00
Traffic commute time index	−1.66	0.10

* *p < 0.05*.

### K-Means Analysis of the Data

**Figure 1:**
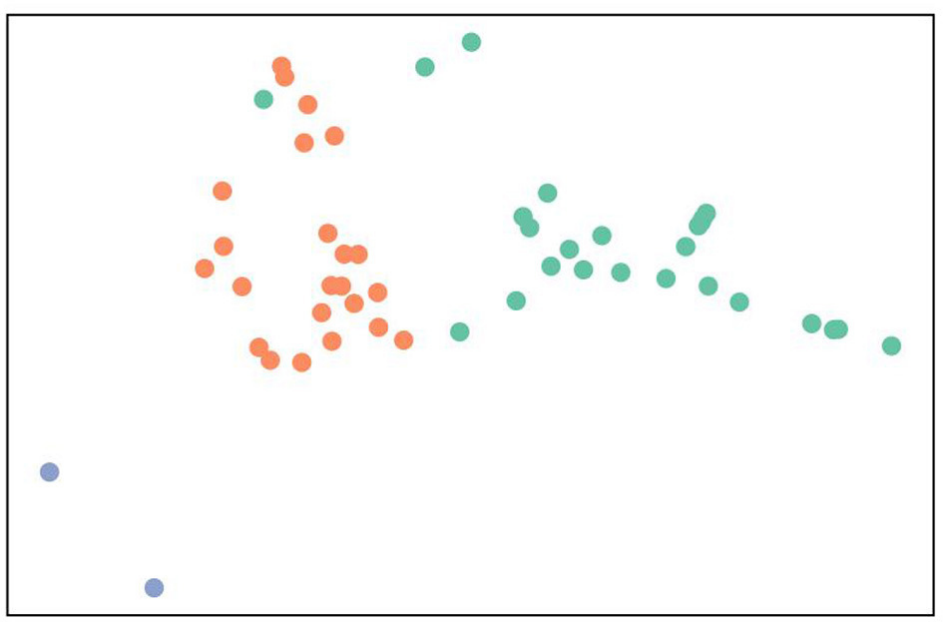
Visual clustering map of 52 countries. Cluster I, Cluster II, and Cluster III was indicated by soft orange dots, lime green, and slightly blue, respectively.

It could be noticed from the results that there were 24, 26, and two countries in Cluster, ClusterII, and Cluster III, respectively. The three Clusters showed different characteristics. In Cluster I, the characteristics of the 24 developing countries were the decline of CLI and CI, and the increase of PI. Except for Croatia, all 25 countries in Cluster II were developed. In addition, developed countries had characteristics of having an increase of HCL and a decline of QLI and SI. Cluster III only included Poland and Chile, which had a decline of PPI and an increase of PPIRI and PI (Table 3 and Figure 1).

**Table 3:** Clustering of 52 countries.

	**Countries**	**Indexes**	**Developed countries**	**Developing countries**
Cluster I	24	dCLI dCI dPI	–	United Arab Emirates, Saudi Arabia, Lithuania, South Africa, Malaysia, Mexico, Hungary, Argentina, India, Serbia, Turkey, Romania, Bulgaria, Thailand, Brazil, Colombia, China, Philippines, Pakistan, Ukraine, Indonesia, Russia, Egypt, Iran
Cluster II	26	dPPI dPPIRI dPI	Switzerland, United States, Germany, Sweden Finland, Denmark, Canada, Australia, Austria, New Zealand, Japan, Norway, Netherlands, United Kingdom, Ireland, France, Belgium, Portugal, Spain, Czech, Republic, South Korea, Israe, Italy, Singapore, Greece	Croatia
Cluster III	2	dQI dSI dHCI	Poland	Chile

### Differences in Nine Indexes Between Developing and Developed Countries

Testing the normality of nine indexes, the distributions of the Difference of Climate Index (dCI) and the Difference of Property Price to Income Ratio Index (dPPIRI) is not normal. To analyze this problem, Mann-Whitney U test was used. The independent sample *t*-test was used to analyze the other seven indexes which were all subjected to normal distribution and variance homogeneity.

Compared with developed countries, under the influence of the COVID-19, the decline of QLI, PPI, and SI in developing countries were relatively small, while the decline of CLI in developing countries was large. The results showed that COVID-19 had a great impact on the QLI, PPI, and SI in developed countries, and a greater impact on CLI in developing countries (Table 4).

**Table 4:** Differences in nine indexes between developing and developed countries.

**Index**	**t/u**	***p***
dQLI^*^	−2.10	0.04
dCI	277.00	0.06
dCLI^*^	3.28	0.00
dHCI	−1.81	0.08
dPI	1.80	0.08
dPPIRI	288.00	0.41
dPPI^*^	−3.42	0.00
dSI^*^	−3.15	0.00
dTCTI	0.95	0.35

* *p < 0.05*.

*d of nine indexes is the forecast of 2020 mid-year minus actual values of 2020 mid-year*.

### The Relationship of Nine Indexes Between Developing and Developed Countries

The severity of the pandemic was expressed by cumulative incidence. For developing countries, the hypothesis was tested with Pearson correlation statistics which showed that there was a significant relationship between the Difference of Cost-of-Living Index (dCLI) and cumulative incidence. The severity of the pandemic impacted on cost of living of residents. However, for developed countries, an inverse correlation between the Difference of Pollution Index (dPI) and cumulative incidence was observed (Table 5). Thus, it could be noted that climate conditions can affect the cumulative incidence.

**Table 5:** The relationship between nine indexes, cumulative incidence, and the score COVID-safety assessment (SCSA) of countries.

**Indexes**	**Developing countries**				**Developed countries**			
	**CuI**		**SCSA**		**CuI**		**SCSA**	
	**Pearson**	***P***	**Pearson**	***P***	**Pearson**	***P***	**Pearson**	***P***
dQLI	−0.06	0.77	−0.34	0.10	0.09	0.68	−0.08	0.71
dCI	0.14	0.51	−0.49	0.01^*^	0.32	0.12	−0.43	0.03^*^
dCLI	0.60	0.00^*^	−0.27	0.20	−0.17	0.43	0.11	0.62
dHCI	−0.12	0.57	−0.57	0.00^*^	0.02	0.93	0.22	0.28
dPI	0.16	0.45	0.06	0.80	−0.53	0.01^*^	0.36	0.08
dPPIRI	0.01	0.97	0.22	0.30	0.03	0.89	0.01	0.97
dPPI	0.12	0.57	0.18	0.39	−0.39	0.06	0.15	0.47
dSI	−0.18	0.41	−0.08	0.73	0.00	0.98	−0.06	0.77
dTCTI	0.08	0.71	−0.19	0.38	−0.10	0.65	0.10	0.62

* *p-value < 0.01*.

*d of nine indexes is the forecast of 2020 mid-year minus actual values of 2020*.

The SCSA reflected the degree of control of the pandemic. For developing countries, there was a significant relationship between dCI, the Difference of Health Care Index (dHCI), and the SCSA. For developed countries, the link between dCI and the SCSA was statistically significant.

## Discussion

Overall, in those countries affected by COVID-19, it can be considered that the quality of life of inhabitants has reduced, the purchasing power of residents of most countries has generally declined, and that the property price to income ratio has increased. Meanwhile, the safety of individuals is threatened (Table 2). From Figure 1 and Table 3, the outstanding feature of each cluster that we can find is the degree of national development. Developed and developing countries show different distribution. These features guide us to conduct further hierarchical analysis on the basis of the level of national development.

In this pandemic, developing countries are less affected in the quality of life, purchasing power, and safety than developed countries (Table 4). The quality of life of people in developed countries have reduced in such a large-scale pandemic. Research shows that young Americans expressed that there was a decline in the quality of life and an increase in psychiatric distress (31). In our results, the per capital purchasing power of developed countries has declined more significantly than that of developing countries. Of course, this is not the case for all developed countries. Another study stated that more support was urgently needed to alleviate the loss of COVID-19 on the more vulnerable people in consideration of the possible duration of social distancing measures and the associated economic impacts (32). This situation is worthy of the attention of the state aid agencies and psychological counseling departments. Contrary to our result, the safety of society in overall developed countries might be worse in these results (33, 34). The difference might be due to our overall comparative analysis of the selected developing and developed countries. Meanwhile, countries that are seriously affected by COVID-19 should pay more attention to social security. Residents should understand and cooperate with the relevant work of the government to put an end to social disorder and reactionary behavior. In our research, the cost of the living level of developing countries is more severely affected than in developed countries. It has been demonstrated that in developing countries, COVID-19 affected the food security status and the stability of food supply chains (35, 36). It is necessary for local governments to pay attention to the quality of living goods and materials with the aim of alleviate the cost of living of residents.

As shown in Table 5, we found that as of 1 July 2020, the COVID-19 notification information shows that in developing countries, the cumulative incidence is positively correlated with the difference of cost-of-living index. There is no doubt that the quality of life of residents have been affected, as it is reflected in medical services, life consumption, work, study, and so on. People in developing countries are faced with heavy loss of family income, affecting their living expenses and quality of life (37, 38). While trying to control the epidemic, relevant governments should also pay attention to the living conditions of all citizens and strive to provide appropriate help and psychological counseling for the people at the bottom of society. In our results, the SCSA is negatively correlated with the health care of residents in developing countries. SCSA is quantified by many aspects of assessment. Moreover, the level of health care could best reflect the SCSA. In India (39), hospital beds and medical equipment were overrun in the face of the huge number of patients infected with COVID-19. People at risk should have confidence and hope for more complete medical facilities. It is important to highlight that good weather conditions are particularly important for the governance and control of the pandemic in our results. Some researches for COVID-19 indicated that temperature, combined with humidity, were the vital risk factors (40, 41). This is consistent with our results. As for the relationship between the climatic conditions and the spread of the virus, it is obviously a huge problem, which is still worthy of further study.

The more developed countries do have better medical facilities and sufficient financial resources, but the degree of development does not mean that they have a faster response and better rational response attitude. In our research, developed countries with aggravated environmental pollution have a higher cumulative incidence (Table 5). There is reason to believe that air pollution has a negative effect of COVID-19 (42–44). Meanwhile, poorer climatic conditions often lead to poor control in countries affected by COVID-19 (45–47). Consequently, countries with better development have the reason to think highly of environmental sanitation in order to reply the diffuse of COVID-19.

Through this research direction and data characteristics, we aim to explore the impact of COVID-19 on social-related levels in lots of countries from a macro perspective. The purpose is not to find differences, but to find changes in types of social-related indexes to further guide the formulation of related strategies and measures. We hope that the pandemic can be triumphed as soon as possible worldwide.

## Conclusion

We analyze the social conditions of many countries from a macro perspective. We utilize the three time series models to forecast values of 2020 mid-year. Then, by comparing the difference between the forecast and actual values, we aim to finding out which index in countries is more affected by COVID-19.

Our article will contribute to countries with different levels of development pay more attention to social changes and take timely and effective measures to adjust social changes while trying to control this pandemic. Moreover, it also will help countries to realize how social changes can emerge if measures regarding social changes and control pandemic crises are not effective and adjusted to the specific needs of the population.

Through our results, we find that COVID-19 has affected the lives of residents in many ways. On one hand, the quality of life, purchasing power, and safety of people have declined. On another, the property price to income ratio has risen. Our further research shows that the changes of social-related indexes in developed and developing countries are different, which is related to their epidemic control measures and severity. For developing countries, the higher CuI has a great impact on quality of life of residents, and the SCSA has a negative correlation with the health care situation. There is a correlation between CuI and environmental pollution during COVID-19. Last but not least, the influence of climate on the development and control of this pandemic deserves more detailed study. Consequently, exploring the changes of social-related indexes is significative, and it is conducive to provide targeted governance strategies for these countries affected by COVID-19.

There are limitations of this study to consider. First, in order to ensure the accuracy of the results and the reliability of the conclusions, the index data of some countries are relatively imperfect. We have to delete the countries where the data is missing and select 52 countries where the data is complete. Second, in this study, we find that climate index is related to the overall performance of epidemic control in both developed and developing countries. However, due to the different research focuses on this study, this important variable has not been further analyzed and discussed in detail. In the follow-up study, we hope to pay more attention to the influence of climate factors on the spread and control of COVID-19 and seek other databases and methods to specifically explore the economy, health care services, and other aspects of different countries are affected by COVID-19.

## Data Availability Statement

The datasets presented in this study can be found in online repositories. The names of the repository/repositories and accession number(s) can be found in the article/Supplementary Material.

## Author Contributions

XG, RC, JJ, and SL had the original idea for the study, and with all co-authors, carried out the design. YY provided valuable insight regarding the methodological approach and organization of the manuscript. YM carried out the statistical analysis. YW, TF, JT, and BS reviewed the consistency of data included in the article. XG and RC wrote the first draft of the manuscript, performed the interpretation of the results, and wrote the final version of article in collaboration with SL and JJ. All authors read and approved the final manuscript.

## Funding

Our research was supported by National Natural Science Foundation of China (Grant No. 11901234), the Fundamental Research Funds for the Central Universities, JLU (Grant No. 93K172020K27), Natural Science Foundation of Jilin Province (Grant No. 20210101481JC), and Shanghai Municipal Science and Technology Major Project (Grant No. 2021SHZDZX0103).

## Conflict of Interest

The authors declare that the research was conducted in the absence of any commercial or financial relationships that could be construed as a potential conflict of interest.

## Publisher's Note

All claims expressed in this article are solely those of the authors and do not necessarily represent those of their affiliated organizations, or those of the publisher, the editors and the reviewers. Any product that may be evaluated in this article, or claim that may be made by its manufacturer, is not guaranteed or endorsed by the publisher.
