# Supplementary material for: Comprehensive Analysis of the COVID-19: Based on the Social-Related Indexes From NUMBEO
Source: Front Public Health. 2022 Apr 28;10:793176. doi: 10.3389/fpubh.2022.793176 (PMC9096155; doi:10.3389/fpubh.2022.793176)
Supplement: Supplementary file 1 [file Data_Sheet_1.doc]

**Supplementary Table 1. The detailed introduction of each index.**

| Index | Introduction |
| --- | --- |
| Quality of Life Index | Quality of Life Index (higher is better) is an estimation of overall quality of life by using an empirical formula which takes into account purchasing power index , pollution index , house price to income ratio lower is better, cost of living index , safety index , health care index traffic commute time index and climate index . |
| Cost of Living Index | Cost of Living Index (Excl. Rent) is a relative indicator of consumer goods prices, including groceries, restaurants, transportation and utilities. Cost of Living Index does not include accommodation expenses such as rent or mortgage. |
| Pollution Index | Pollution Index is an estimation of the overall pollution in the city. The biggest weight is given to air pollution, than to water pollution/accessibility, two main pollution factors. Small weight is given to other pollution types. |
| Price to Income Ratio Index | Price to Income Ratio is the basic measure for apartment purchase affordability (lower is better). It is generally calculated as the ratio of median apartment prices to median familial disposable income, expressed as years of income (although variations are used also elsewhere). |
| Traffic Index | Traffic Index is a composite index of time consumed in traffic due to job commute, estimation of time consumption dissatisfaction, CO2 consumption estimation in traffic and overall inefficiencies in the traffic system. |
| Climate Index | Climate Index is an estimation of the climate likability of a given city or a country. It is in the range [-100, +100] (higher is better). Cities with climate index 100 have moderate temperatures and low humidity and no other major weather condition which is usually not preferred by most people. However, some persons prefer colder climate while others prefer warmer climates and some people are fine with humid conditions, so this index is general guidance, which shall not be blindly considered. |
| Health Care Index | Health Care Index is an estimation of the overall quality of the health care system, health care professionals, equipment, staff, doctors, cost, etc. |
| Safety Index | If the city has a high safety index, it is considered very safe. |
| Purchasing Power Index | Local Purchasing Power shows relative purchasing power in buying goods and services in a given city for the average net salary in that city. |

**
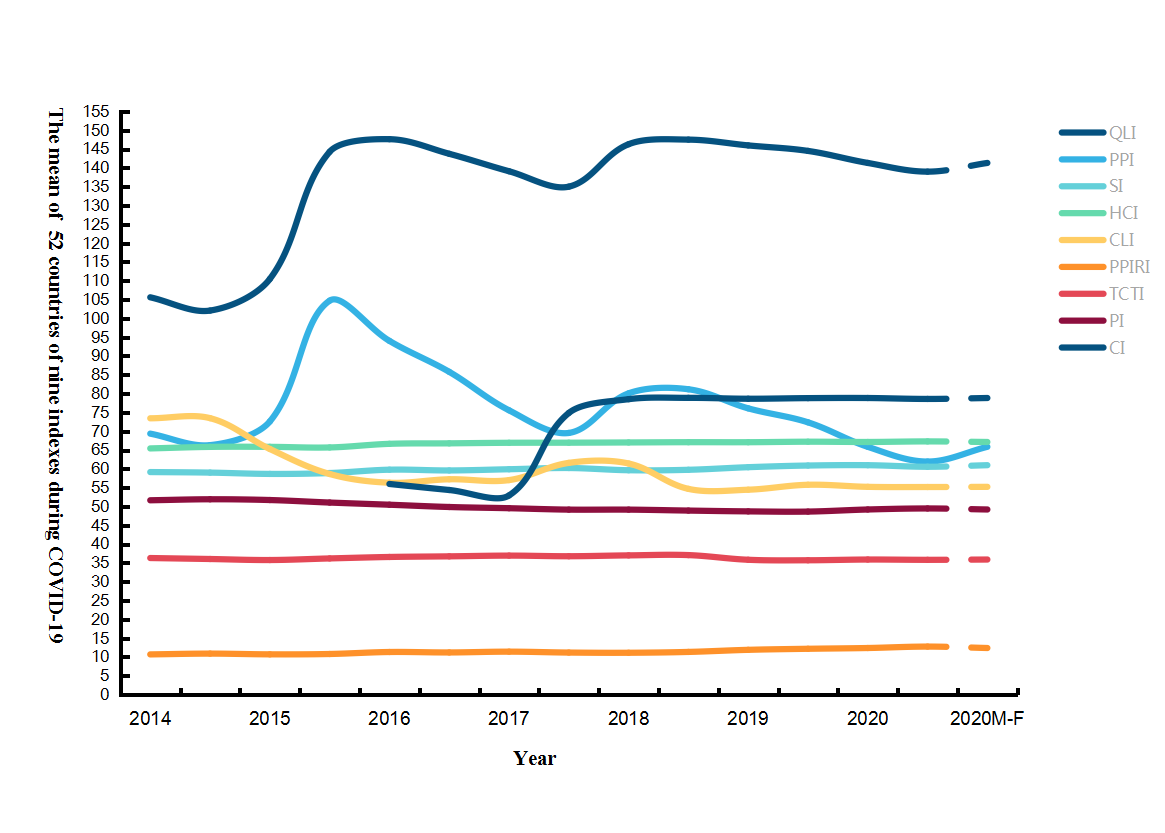
**

**Supplementary Figure 1.** The mean of 52 countries of nine indexes during COVID-19 (including actual and forecast values).

Note: 2020M-F：the forecast values of 2020 mid-year; QLI: Quality of Life Index; CI: Climate Index; CLI: Cost of Living Index; HCI: Health Care Index; PI: Pollution Index; PPIRI: Property Price to Income Ratio Index; PPI: Purchasing Power Index; SI: Safety Index; TCTI: Traffic Commute Time Index

**Supplementary Table 2.** The actual value and forecast value of 52 countries in 2020 mid-year.

| Country | QLI | | CI | | CLI | | HCI | | PI | | PPIRI | | PPI | | SI | | TCTI | |
| --- | --- | --- | --- | --- | --- | --- | --- | --- | --- | --- | --- | --- | --- | --- | --- | --- | --- | --- |
|  | AV | FV | AV | FV | AV | FV | AV | FV | AV | FV | AV | FV | AV | FV | AV | FV | AV | FV |
| Switzerland | 190.92 | 192.01 | 80.05 | 79.24 | 125.69 | 122.40 | 73.10 | 72.44 | 21.89 | 22.39 | 8.52 | 8.68 | 114.83 | 119.53 | 78.42 | 78.40 | 28.93 | 29.09 |
| United States | 169.78 | 172.11 | 76.69 | 77.54 | 72.47 | 71.05 | 69.20 | 69.27 | 38.17 | 36.88 | 3.72 | 3.52 | 108.29 | 109.52 | 52.30 | 52.80 | 33.02 | 32.89 |
| Germany | 177.25 | 179.78 | 83.15 | 83.00 | 66.34 | 65.26 | 73.35 | 73.32 | 29.28 | 29.03 | 9.35 | 9.38 | 97.41 | 102.36 | 64.86 | 65.19 | 31.40 | 30.98 |
| Sweden | 172.18 | 175.95 | 73.97 | 73.97 | 72.31 | 69.85 | 69.15 | 69.23 | 19.05 | 18.09 | 8.98 | 9.27 | 94.04 | 101.73 | 52.57 | 52.93 | 30.29 | 30.49 |
| Finland | 186.40 | 190.22 | 59.21 | 58.87 | 72.71 | 70.29 | 75.96 | 75.79 | 11.79 | 11.55 | 8.69 | 8.35 | 93.94 | 99.93 | 74.47 | 76.68 | 29.41 | 29.90 |
| Denmark | 192.53 | 192.67 | 81.80 | 81.80 | 85.02 | 83.00 | 80.17 | 80.00 | 21.52 | 21.33 | 6.96 | 7.45 | 101.27 | 100.88 | 73.91 | 74.90 | 28.93 | 28.85 |
| Canada | 158.88 | 163.47 | 52.69 | 50.57 | 66.18 | 67.62 | 71.77 | 71.58 | 27.97 | 27.83 | 8.18 | 7.97 | 83.22 | 95.09 | 59.36 | 60.33 | 33.84 | 33.87 |
| Australia | 185.03 | 186.21 | 94.20 | 92.70 | 75.89 | 73.54 | 77.81 | 77.38 | 23.22 | 23.46 | 7.44 | 7.52 | 103.38 | 107.31 | 58.33 | 58.64 | 35.00 | 34.73 |
| United Arab Emirates | 155.41 | 156.67 | 45.23 | 45.23 | 62.98 | 61.98 | 67.92 | 67.04 | 50.66 | 51.15 | 5.14 | 5.29 | 86.06 | 91.58 | 84.55 | 84.30 | 36.63 | 36.85 |
| Austria | 181.68 | 182.50 | 78.22 | 77.30 | 71.78 | 70.38 | 78.56 | 78.73 | 21.65 | 22.19 | 10.85 | 10.88 | 79.38 | 82.38 | 75.57 | 76.27 | 25.79 | 26.27 |
| New Zealand | 178.22 | 181.02 | 95.46 | 95.46 | 71.83 | 72.53 | 73.82 | 73.81 | 23.52 | 23.40 | 8.66 | 8.52 | 87.22 | 92.66 | 57.81 | 59.07 | 30.81 | 31.10 |
| Japan | 163.23 | 167.99 | 84.79 | 84.79 | 86.22 | 83.35 | 80.23 | 81.14 | 39.34 | 39.59 | 13.28 | 13.00 | 78.88 | 87.28 | 78.33 | 79.34 | 39.41 | 39.15 |
| Norway | 174.55 | 175.19 | 71.37 | 71.37 | 96.8 | 101.43 | 74.51 | 74.36 | 20.14 | 20.35 | 8.68 | 8.77 | 83.40 | 88.38 | 65.38 | 64.57 | 26.62 | 26.99 |
| Netherlands | 184.18 | 183.67 | 87.53 | 87.61 | 74.63 | 73.75 | 75.68 | 74.65 | 26.11 | 27.41 | 7.45 | 7.51 | 86.76 | 90.73 | 72.85 | 72.38 | 28.15 | 29.43 |
| United Kingdom | 161.20 | 162.71 | 87.77 | 87.62 | 65.67 | 67.28 | 74.88 | 74.46 | 40.62 | 40.56 | 9.63 | 10.09 | 86.98 | 91.73 | 55.46 | 56.29 | 34.51 | 34.53 |
| Ireland | 150.16 | 153.53 | 89.13 | 89.13 | 78.07 | 75.91 | 52.27 | 51.89 | 34.29 | 33.99 | 7.75 | 7.49 | 74.58 | 80.88 | 54.32 | 54.57 | 38.07 | 37.68 |
| Saudi Arabia | 148.94 | 150.56 | 45.98 | 45.98 | 49.15 | 48.34 | 60.42 | 59.11 | 64.34 | 65.09 | 2.72 | 2.79 | 93.84 | 100.00 | 73.32 | 73.82 | 28.45 | 28.61 |
| France | 150.68 | 153.95 | 89.78 | 90.25 | 76.34 | 74.14 | 80.68 | 79.99 | 43.64 | 43.56 | 14.07 | 13.04 | 76.21 | 80.36 | 52.63 | 53.21 | 35.04 | 34.76 |
| Belgium | 149.75 | 153.47 | 86.03 | 85.99 | 73.59 | 71.78 | 74.80 | 74.34 | 53.66 | 52.94 | 6.91 | 6.91 | 80.12 | 86.28 | 54.71 | 56.02 | 36.43 | 36.15 |
| Portugal | 162.46 | 162.91 | 97.72 | 97.31 | 49.52 | 49.52 | 71.88 | 71.88 | 30.38 | 30.89 | 12.52 | 12.70 | 47.10 | 49.43 | 70.17 | 70.37 | 30.13 | 30.00 |
| Spain | 167.05 | 169.82 | 93.98 | 94.19 | 55.27 | 53.77 | 78.69 | 78.88 | 40.37 | 39.99 | 9.55 | 9.37 | 67.73 | 72.03 | 67.67 | 68.04 | 29.38 | 29.10 |
| Czech Republic | 154.70 | 156.24 | 77.13 | 77.13 | 45.05 | 46.15 | 75.18 | 74.62 | 39.46 | 40.23 | 16.43 | 15.42 | 59.28 | 62.82 | 74.60 | 74.48 | 29.71 | 29.65 |
| Lithuania | 159.77 | 159.42 | 69.86 | 67.76 | 44.85 | 44.28 | 70.51 | 69.49 | 27.91 | 28.80 | 11.09 | 10.90 | 55.57 | 57.85 | 66.12 | 66.94 | 25.65 | 26.08 |
| South Korea | 136.27 | 139.02 | 68.39 | 68.39 | 75.93 | 78.18 | 82.26 | 81.97 | 62.25 | 62.48 | 19.41 | 17.37 | 81.67 | 85.21 | 72.67 | 71.98 | 39.86 | 39.57 |
| South Africa | 132.39 | 131.97 | 95.25 | 95.25 | 37.52 | 42.87 | 64.16 | 64.14 | 57.12 | 57.30 | 4.24 | 3.93 | 73.52 | 73.61 | 22.71 | 22.51 | 39.42 | 39.43 |
| Croatia | 156.77 | 159.01 | 89.89 | 89.05 | 56.31 | 49.70 | 63.75 | 62.68 | 30.07 | 30.46 | 13.91 | 13.73 | 43.25 | 50.42 | 75.33 | 75.29 | 28.14 | 29.11 |
| Poland | 125.20 | 141.83 | 76.03 | 76.14 | 39.46 | 40.04 | 51.96 | 61.01 | 63.09 | 54.46 | 13.20 | 11.38 | 52.30 | 59.61 | 66.87 | 71.50 | 32.16 | 31.72 |
| Israel | 146.89 | 149.94 | 93.78 | 93.78 | 82.52 | 81.15 | 73.40 | 73.29 | 57.48 | 57.25 | 13.47 | 12.83 | 73.94 | 78.09 | 69.56 | 70.40 | 36.08 | 35.91 |
| Italy | 138.97 | 140.76 | 91.64 | 92.27 | 68.95 | 67.26 | 66.95 | 66.59 | 55.65 | 55.63 | 9.81 | 9.71 | 61.80 | 65.59 | 55.76 | 55.74 | 34.3 | 34.42 |
| Malaysia | 117.42 | 118.44 | 59.21 | 57.92 | 38.09 | 39.12 | 68.87 | 68.10 | 62.81 | 63.18 | 10.00 | 9.94 | 59.12 | 64.49 | 41.45 | 41.16 | 37.11 | 37.03 |
| Singapore | 142.25 | 144.39 | 57.45 | 57.45 | 81.13 | 81.10 | 70.97 | 70.84 | 33.30 | 33.48 | 21.67 | 22.31 | 82.61 | 88.96 | 68.47 | 69.43 | 41.18 | 41.31 |
| Mexico | 120.65 | 118.55 | 86.29 | 86.29 | 30.93 | 35.72 | 72.11 | 70.12 | 63.29 | 66.1 | 10.69 | 10.67 | 38.17 | 41.81 | 46.69 | 46.03 | 39.20 | 39.39 |
| Hungary | 128.40 | 128.16 | 79.48 | 79.48 | 40.09 | 40.85 | 48.05 | 47.80 | 48.66 | 48.29 | 13.82 | 14.77 | 46.30 | 47.55 | 64.77 | 64.92 | 35.91 | 35.78 |
| Argentina | 114.21 | 115.31 | 98.28 | 98.28 | 31.18 | 32.95 | 69.16 | 69.25 | 50.61 | 50.67 | 24.09 | 24.53 | 43.38 | 47.22 | 37.74 | 38.23 | 42.99 | 43.08 |
| India | 105.86 | 108.63 | 64.74 | 64.87 | 24.12 | 24.58 | 66.21 | 67.13 | 79.56 | 78.87 | 11.34 | 11.38 | 50.55 | 54.30 | 55.58 | 56.68 | 46.83 | 46.99 |
| Greece | 131.51 | 133.07 | 94.18 | 94.18 | 57.50 | 55.67 | 56.64 | 56.21 | 52.33 | 52.55 | 10.70 | 10.60 | 40.75 | 43.68 | 58.70 | 59.68 | 33.71 | 33.84 |
| Chile | 102.02 | 119.76 | 90.21 | 90.21 | 45.43 | 43.62 | 64.38 | 65.44 | 79.74 | 65.78 | 17.94 | 14.93 | 35.55 | 42.50 | 50.40 | 54.77 | 35.16 | 35.44 |
| Serbia | 116.08 | 116.30 | 83.23 | 83.23 | 36.98 | 35.72 | 51.74 | 51.27 | 61.21 | 60.32 | 17.90 | 18.94 | 36.04 | 36.72 | 62.27 | 62.59 | 31.02 | 30.43 |
| Turkey | 126.40 | 127.10 | 93.26 | 93.26 | 32.44 | 34.69 | 70.36 | 69.80 | 66.22 | 67.35 | 8.48 | 7.81 | 38.04 | 40.85 | 60.50 | 60.51 | 44.86 | 44.65 |
| Romania | 131.69 | 132.44 | 77.69 | 77.62 | 36.23 | 35.31 | 55.46 | 55.06 | 58.79 | 58.42 | 10.96 | 11.33 | 46.70 | 48.86 | 72.18 | 72.36 | 34.75 | 34.75 |
| Bulgaria | 127.14 | 129.80 | 82.76 | 82.76 | 38.39 | 36.70 | 56.20 | 55.40 | 65.43 | 65.33 | 9.11 | 8.52 | 44.11 | 49.37 | 61.45 | 61.50 | 29.45 | 29.39 |
| Thailand | 101.64 | 101.88 | 69.45 | 69.45 | 48.97 | 49.77 | 78.04 | 77.95 | 75.26 | 75.07 | 21.59 | 22.26 | 33.02 | 35.45 | 59.99 | 59.52 | 38.54 | 38.23 |
| Brazil | 105.28 | 105.65 | 94.10 | 97.16 | 30.99 | 40.22 | 56.87 | 56.29 | 54.67 | 54.98 | 16.47 | 16.41 | 30.36 | 32.81 | 31.69 | 31.12 | 41.63 | 41.70 |
| Colombia | 101.70 | 105.83 | 86.04 | 96.69 | 29.02 | 30.66 | 66.80 | 67.24 | 62.44 | 62.83 | 19.29 | 19.65 | 27.92 | 31.12 | 45.06 | 45.21 | 47.16 | 47.49 |
| China | 104.96 | 102.81 | 78.17 | 79.19 | 39.51 | 40.04 | 66.62 | 64.48 | 79.88 | 80.77 | 28.40 | 29.06 | 60.44 | 60.88 | 68.82 | 68.17 | 41.44 | 41.81 |
| Philippines | 79.18 | 85.37 | 61.11 | 60.81 | 39.25 | 37.63 | 67.52 | 67.47 | 74.24 | 74.28 | 29.92 | 24.60 | 21.38 | 23.48 | 57.78 | 57.84 | 44.60 | 44.63 |
| Pakistan | 105.09 | 105.44 | 71.93 | 71.93 | 21.00 | 21.98 | 60.69 | 60.59 | 73.48 | 74.25 | 12.46 | 11.98 | 28.39 | 30.57 | 55.82 | 55.92 | 37.77 | 38.56 |
| Ukraine | 105.26 | 104.77 | 71.14 | 71.42 | 30.05 | 33.18 | 52.69 | 52.33 | 65.14 | 65.08 | 11.88 | 12.23 | 30.90 | 31.80 | 51.16 | 51.15 | 38.32 | 38.65 |
| Indonesia | 91.63 | 97.47 | 68.48 | 74.15 | 36.86 | 37.27 | 60.51 | 60.48 | 66.90 | 66.56 | 21.27 | 18.88 | 22.19 | 25.05 | 53.94 | 54.16 | 43.37 | 43.11 |
| Russia | 101.57 | 102.31 | 39.51 | 40.36 | 33.66 | 39.21 | 58.01 | 57.59 | 62.58 | 62.79 | 11.30 | 10.77 | 35.96 | 38.94 | 59.40 | 58.88 | 45.00 | 45.30 |
| Egypt | 86.78 | 86.54 | 91.98 | 91.98 | 29.55 | 29.54 | 46.29 | 45.84 | 84.69 | 85.65 | 13.42 | 13.01 | 21.09 | 22.41 | 53.35 | 53.08 | 49.34 | 49.78 |
| Iran | 70.32 | 74.14 | 70.99 | 70.99 | 38.47 | 39.01 | 52.23 | 51.70 | 76.69 | 77.45 | 29.83 | 25.11 | 21.88 | 22.69 | 51.09 | 50.75 | 47.44 | 48.01 |

**QLI**: Quality of Life Index; **CI**: Climate Index; **CLI**: Cost of Living Index; **HCI**: Health Care Index; **PI**: Pollution Index; **PPIRI**: Property Price to Income Ratio Index; **PPI**: Purchasing Power Index; **SI**: Safety Index; **TCTI**: Traffic Commute Time Index

**AV**: Actual Value; **FV**: Forecast Value
